# Diagnóstico por Imagem: Origem Anômala da ACE Saindo do Tronco da Artéria Pulmonar

**DOI:** 10.36660/abc.20180207

**Published:** 2020-05-11

**Authors:** Haisong Bu, Tianli Zhao

**Affiliations:** 1 Second Xiangya Hospital Hunan China Second Xiangya Hospital, Changsha, Hunan – China; 2 Second Xiangya Hospital Central South University Department of Cardiovascular Surgery Changsha China Second Xiangya Hospital - Central South University - Department of Cardiovascular Surgery, Changsha – China

**Keywords:** Cardiopatias Congênitas/cirurgia, Insuficiência da Valva Mitral/cirurgia, Isquemia Miocárdica, Diagnóstico por Imagem, Imagem por Ressonância Magnética, Artéria Pulmonar/anormalidades, Ecocardiografia/métodos

## Introdução

A origem anômala da artéria coronária esquerda saindo do tronco da artéria pulmonar (ALCAPA) é uma anomalia congênita rara, com mortalidade de 90% até o 1^o^ ano de idade sem intervenção cirúrgica.^1^ Atualmente, o procedimento de escolha para correção da ALCAPA depende do estabelecimento de um sistema arterial coronariano duplo por reimplante direto da artéria coronária esquerda anômala (ACE) na aorta ascendente. Entretanto, variações anatômicas da origem da ACE anômala costumam dificultar esse objetivo, principalmente em pacientes submetidos à reoperação.

A regurgitação mitral (RM) isquêmica crônica evolui como consequência da coronariopatia na ausência de alterações de folheto primário ou patologia cordal:^2^ a cardiopatia isquêmica causa remodelamento da geometria ventricular esquerda, deslocamento dos músculos papilares, deslocamento dos músculos papilares, *tethering* dos folhetos e dilatação anular, levando à insuficiência mitral funcional. O desfecho desses pacientes representa um problema desafiador tanto para cardiologistas quanto para cirurgiões cardiovasculares. De fato, o papel da cirurgia da válvula mitral (CVM) associado à revascularização da artéria coronária ainda é controverso.

## Relato de caso 1

**Figura 1:**
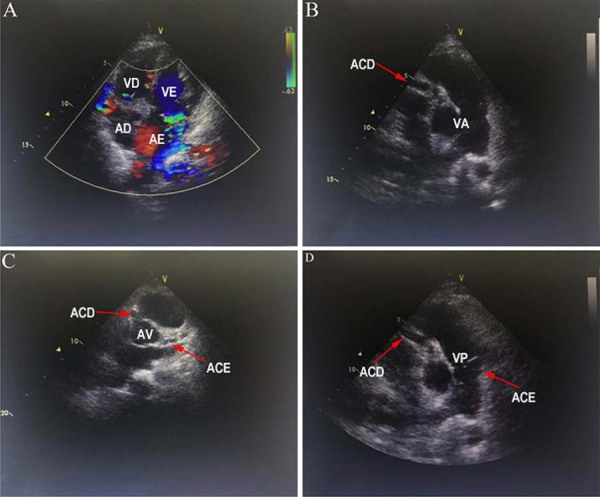
- Ecocardiograma transtorácico mostrou regurgitação mitral moderada (A) e o diâmetro da ACD mostrou-se aumentado na extremidade proximal (B, setas), e a ACE não vinha do seio da coroa esquerda (C, setas), estando inserida na artéria pulmonar (D, setas). VD: ventrículo direito; AD: átrio direito; VE: ventrículo esquerdo; AE: átrio esquerdo; VA: valva aórtica; ACD: artéria coronária direita; ACE: artéria coronária esquerda; VP: valva pulmonar.

**Figura 2:**
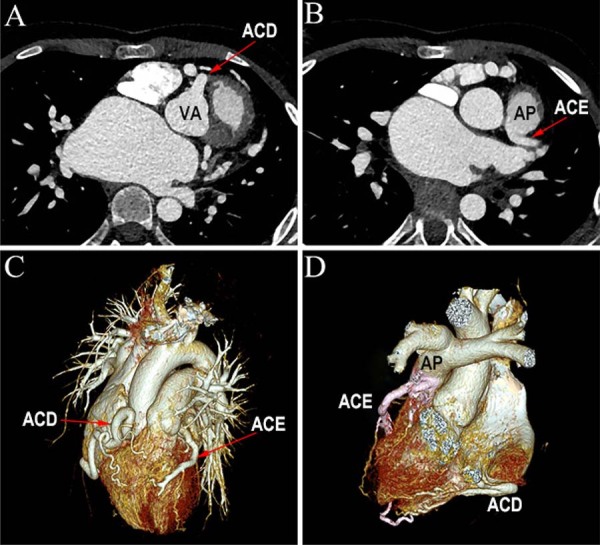
- A imagem da angiotomografia computadorizada mostrou ACD gigante e torcida (A e C, setas) e ALCAPA (B e D, setas). VA: valva aórtica; ACD: artéria coronária direita; ACE: artéria coronária esquerda; AP: artéria pulmonar.

**Figura 3:**
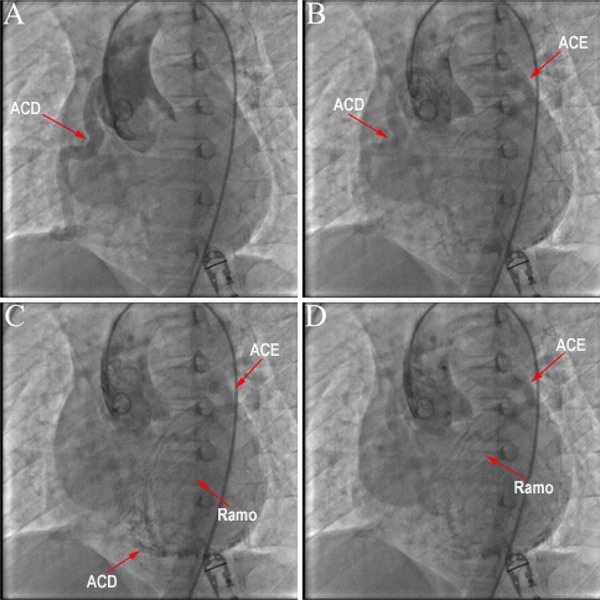
- A angiografia da aorta ascendente mostrou ACD dilatada e torcida e ALCAPA (setas A e B), além do fenômeno de roubo de fluxo sanguíneo (C e D, setas). ACD: artéria coronária direita; ACE: artéria coronária esquerda.

Menino de 11 anos de idade de uma aldeia remota no sul da China, valvoplastia mitral prévia sem melhora sintomática significativa. Ecocardiograma transtorácico (ETT) mostrou átrio esquerdo aumentado de 61 mm, RM moderada ( Figura 1A ) e fração de ejeção de 60%. O diâmetro da artéria coronária direita (ACD) encontrava-se aumentado, com 7 mm, na extremidade proximal ( Figura 1B ), e a ACE não saía do seio coronariano esquerdo ( Figura 1C ), inserida na artéria pulmonar através de uma fístula de 5 mm ( Figura 1D ). A angiotomografia tridimensional computadorizada (ATC) da artéria coronária mostrava ALCAPA ( Figura 2B e D ) com ACD gigante e torcida ( Figuras 2A e C ). Angiografia da aorta ascendente mostrava ACD dilatada e torcida ( Figuras 3A e B ) e ALCAPA. A direção do fluxo sanguíneo coronariano era ACD-ramo comunicante-ACE ( Figura 3C e D ) e, portanto, havia roubo de fluxo coronário.

## Relato de caso 2

**Figura 4:**
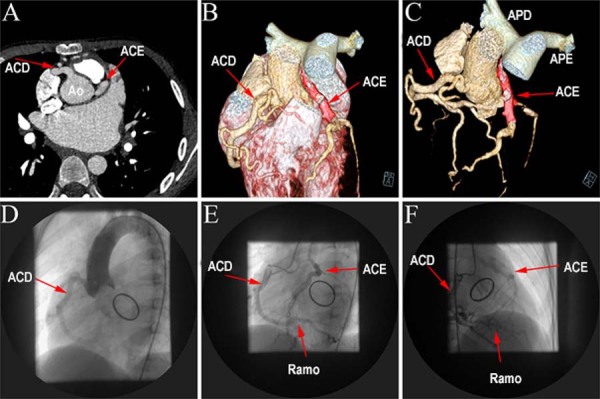
- A imagem da angiotomografia computadorizada mostrou ACD gigante (A, B e C, setas) e ALCAPA (C, setas); A angiografia da aorta ascendente mostrou apenas ACD dilatada e torcida (D, setas) e um fenômeno de roubo sanguíneo (E e F, setas). Ao: aorta; ACD: artéria coronária direita; ACE: artéria coronária esquerda; APE: artéria pulmonar esquerda; APD: artéria pulmonar direita.

Menino de 9 anos de idade, chinês, troca valvar mitral prévia, feita sete anos antes, apresentou febre recorrente e dispneia aos esforços e foi encaminhado ao nosso departamento. ETT mostrava átrio esquerdo aumentado de 58 mm, vazamento parabasilar mitral moderado e fração de ejeção de 62%. O diâmetro da ACE encontrava-se aumentado, com 5 mm, na extremidade proximal, e a ACE não saía do seio da coroa esquerda. ATC tridimensional da artéria coronária mostrava ALCAPA ( Figuras 4B e C ) com ACD gigante ( Figuras 4A, B e C ). Angiografia da aorta ascendente mostrou ACD dilatada e torcida ( Figura 4D ). Havia fenômeno de roubo de fluxo coronário. A direção do fluxo coronário era da ACD-ramo comunicante-ACE-artéria pulmonar ( Figuras 4E e F ).

## Resultados e conclusão

ALCAPA é uma anomalia congênita rara, com mortalidade de 90% no 1^o^ ano de idade sem intervenção cirúrgica. Noventa por cento dos pacientes apresentam, no primeiro ano de vida, sinais e sintomas de insuficiência cardíaca ou morte súbita cardíaca secundária a isquemia miocárdica crônica.^3^ Os sobreviventes adultos, no entanto, são assintomáticos ou apresentam dispneia, angina, RM, isquemia miocárdica ou arritmia ventricular, hipertensão pulmonar e morte súbita. Esse fato é contrastante com a apresentação clínica de isquemia miocárdica e infarto do miocárdio (palpitações, angina e fadiga) em crianças e má evolução ponderal, irritabilidade, transpiração excessiva e apatia em bebês.^4 , 5^

Durante a infância, há diminuição da pressão pulmonar e declínio nos níveis de oxigênio, levando à diminuição da perfusão coronária e isquemia, principalmente durante a alimentação ou choro, quando a demanda miocárdica de oxigênio aumenta. A isquemia miocárdica crônica leva ao comprometimento da função não apenas do miocárdio, mas também do aparelho valvar mitral com insuficiência cardíaca e, posteriormente, regurgitação valvar mitral. Se essa fase for tolerada, ocorrem alterações compensatórias ao longo do tempo e o miocárdio é remodelado durante a infância. Devido ao desenvolvimento de colaterais intercoronarianas partindo da ACD, cada vez maior, o fornecimento de suprimento colateral à ACE leva a uma reversão do fluxo da coronária esquerda anômala para a artéria pulmonar.^3^ Finalmente, existem vasos colaterais excessivos que levam ao desvio de sangue da ACD via colaterais para a ACE e na artéria pulmonar durante a fase adulta.^3 , 5^ Isso é visto como um exemplo do fenômeno de roubo de fluxo sanguíneo.

O ecocardiograma é a base da ferramenta de diagnóstico não invasiva durante a triagem precoce, que descreve a origem anormal da ACE com jato anormal, dilatação ventricular esquerda, ACD dilatada, enchimento retrógrado, hipocinesia leve da parede anterior, presença de hiperecogenicidade do endocárdio e/ou músculos papilares, diâmetro da ACD como proporção do diâmetro do anel da aorta, etc.^4^

Tradicionalmente, o diagnóstico de ALCAPA é feito por angiografia coronária. Nos últimos anos, a angiotomografia computadorizada de coronárias (ATCC) surgiu como o padrão de referência para a identificação e caracterização de anomalias das artérias coronárias. A ATCC permite um diagnóstico preciso não invasivo, descrevendo a origem e o curso das artérias coronárias. Além disso, oferece uma avaliação tridimensional das relações anatômicas entre as artérias coronárias e as estruturas adjacentes,^6^ possibilitando vistas em corte das estruturas cardíacas sob vários ângulos. Portanto, poderia ser considerada a modalidade de imagem de escolha para delinear de forma não invasiva a anatomia dos vasos coronários. Além disso, desempenha um papel importante no planejamento da intervenção cirúrgica, podendo ser uma valiosa ferramenta de acompanhamento pós-operatório para os pacientes.^7^

O crescente uso da ressonância magnética cardíaca (RMC) não apenas aumentou o rendimento diagnóstico, como também possibilitou uma melhor avaliação das consequências da hipoperfusão miocárdica e de defeitos congênitos associados. A presença de dilatação do ventrículo esquerdo, cicatrização subendocárdica e alterações regionais da motilidade segmentar são indicadores de isquemia crônica. E a presença de realce subendocárdico tardio pode ser observada nas imagens de RMC, sugerindo isquemia subendocárdica crônica, sendo considerado um sinal muito importante, principalmente em pacientes assintomáticos.^3^ A correção cirúrgica deve ser fortemente considerada se esse achado estiver presente. A RMC tem sido cada vez mais utilizada em vários outros estudos para orientar as decisões diagnósticas e terapêuticas em pacientes com ALCAPA.

O reimplante na aorta é o único reparo anatômico verdadeiro, mas os benefícios da CVM no momento da operação da ALCAPA devem ser pesados contra os efeitos do *bypass* prolongado em caso de ventrículo esquerdo já isquêmico.^3^ Após a valvoplastia mitral/troca valvar e transplante de ACE, não foram observados sintomas de fenômeno de roubo de fluxo sanguíneo, e a cintilografia de perfusão miocárdica não mostrou alterações isquêmicas. O ecocardiograma pós-operatório, 7 dias após o procedimento, mostrou que a ACE se origina da aorta, com boa visualização dos óstios coronários, diminuição do átrio esquerdo e do ventrículo esquerdo, sem RM e fração de ejeção ventricular aumentada para 72%.

Portanto, a dispneia e a RM induzidas pelo exercício provavelmente ocorreram devido ao fenômeno de roubo de artéria coronária devido à origem anômala de uma artéria coronária. Este relato destaca a essência do aumento dos índices de diagnóstico pré-operatório na aldeia remota da China. Pacientes com insuficiência mitral moderada ou importante sem outras causas aparentes, com dilatação do ventrículo esquerdo e possível presença de hiperecogenicidade do endocárdio e/ou músculos papilares, sem boa visualização dos óstios coronários (ou com suspeita de origem anômala ou dilatação de artéria coronária) foram submetidos à angiotomografia tridimensional computadorizada ou angiotomografia cardíaca.
