# Supplementary material for: Diagnóstico por Imagem: Origem Anômala da ACE Saindo do Tronco da Artéria Pulmonar
Source: Arq Bras Cardiol. 2020 May 11;114(4 Suppl 1):4–7. [Article in Portuguese] doi: 10.36660/abc.20180207 (PMC8149114; doi:10.36660/abc.20180207)
Supplement: Translation

# Translation: Image Diagnosis: An Anomalous Origin of Left Coronary Artery from the Pulmonary Artery

**Authors:** Haisong Bu, Tianli Zhao

**Affiliations:** 1 China Second Xiangya Hospital, Changsha, Hunan – China; 2 China Second Xiangya Hospital - Central South University - Department of Cardiovascular Surgery, Changsha – China

**Keywords:** Heart Defects,Congenital/surgery, Insufficiency Mitral Valve/surgery, Myocardial Ischemia, Diagnostic Imaging, Magnetic Resonance Imaging/methods, Pulmonary Artery/abnormalities, Echocardiography/methods

## Introduction

Anomalous origin of the left coronary artery from the pulmonary artery (ALCAPA) is a rare congenital anomaly with a mortality of 90% by 1 year of age without surgical intervention.^1^ Nowadays the procedure of choice for correction of ALCAPA depends on the establishment of a dual coronary artery system by direct reimplantation of the anomalous left coronary artery (LCA) into the ascending aorta. However, anatomic variations of the origin of the anomalous LCA often make this aim difficult to achieve, especially in patients undergoing reoperation.

Chronic ischemic mitral regurgitation (MR) develops as a consequence of coronary artery disease in the absence of primary leaflet abnormalities or chordal pathology:^2^ ischemic cardiopathy causes remodeling of left ventricular geometry, displacement of papillary muscles, leaflet tethering and annular dilatation, leading to functional mitral insufficiency. The outcome of these patients represents a challenging problem for both cardiologists and cardiac surgeons. In fact, the role of mitral valve surgery (MVS) associated with coronary artery revascularization is still debated.

## Case report 1

11-year-old boy from a remote village in southern China, s/p mitral valvuloplasty without significant symptomatic improvement. Transthoracic echocardiography (TTE) showed an enlarged left atrium of 61 mm, moderate MR ( Figure 1A ), and an ejection fraction of 60%. The right coronary artery (RCA) diameter was increased to 7 mm at the proximal end ( Figure 1B ), and the LCA was not from a left coronary sinus ( Figure 1C ) and inserted into the pulmonary artery through a 5-mm fistula ( Figure 1D ). Three-dimensional coronary artery computed tomography angiography (CTA) showed an ALCAPA ( Figure 2B and D ) with a giant and twisted RCA ( Figure 2A and C ). Ascending aorta angiography showed dilated and twisted RCA ( Figure 3A and B ) and ALCAPA. The direction of coronary blood flow was RCA-communicating branch-LCA-pulmonary artery ( Figure 3C and D ) and hence there was a coronary steal.

**Figure 1:** - Transthoracic echocardiography showed moderate mitral regurgitation (A) and the RCA diameter was increased at the proximal end (B, arrows), and the LCA was not from left crown sinus (C, arrows) and inserted into the pulmonary artery (D, arrows). RV: right ventricle; RA: right atrium; LV: left ventricle; LA: left atrium; AV: aortic valve; RCA: right coronary artery; LCA: left coronary artery; PV: pulmonary valve.

**Figure 2:** – Computed tomography angiography scan image showed a giant and twisted RCA (A and C, arrows) and ALCAPA (B and D, arrows). AV: aortic valve; RCA: right coronary artery; LCA: left coronary artery; PA: pulmonary artery.

**Figure 3:** - Ascending aorta angiography showed dilated and twisted RCA and ALCAPA (A and B, arrows) as well as a phenomenon of stealing blood (C and D, arrows). RCA: right coronary artery; LCA: left coronary artery.

## Case report 2

A 9-year-old Chinese boy status post mitral valve replacement for about seven years presented with repeated fever and exertional dyspnea and referred to our department. TTE showed an enlarged left atrium of 58mm, moderate mitral parabasilar leak, and an ejection fraction of 62%. The RCA diameter was increased to 5 mm at the proximal end, and the LCA was not from left crown sinus. Three-dimensional coronary artery CTA showed an ALCARPA ( Figure 4B and C ) with a giant RCA ( Figure 4A, B and C ). Ascending aorta angiography showed dilated and twisted RCA ( Figure 4D ). There was a phenomenon of stealing blood. The direction of coronary blood flow was from RCA-communicating branch-LCA-pulmonary artery ( Figure 4E and F ).

**Figure 4:** - Computed tomography angiography scan image showed a giant RCA (A, B and C, arrows) and ALCARPA (C, arrows); Ascending aorta angiography only showed dilated and twisted RCA (D, arrows) and a phenomenon of stealing blood (E and F, arrows). Ao: aorta; RCA: right coronary artery; LCA: left coronary artery; LPA: left pulmonary artery; RPA: right pulmonary artery.

## Result and conclusion

ALCAPA is a rare congenital anomaly with a mortality of 90% by 1 year of age without surgical intervention. Ninety percent of patients present in the first year of life with signs and symptoms of heart failure or sudden cardiac death secondary to chronic myocardial ischemia.^3^ Adult survivors, however, are either asymptomatic or present with dyspnea, angina, MR, myocardial ischemia, or ventricular arrhythmia, pulmonary hypertension, and sudden death. This contrasts with the clinical presentation of myocardial ischemia and infarction (palpitations, angina and fatigue) in children, and failure to thrive, irritability, excessive sweating, and listlessness in babies.^4 , 5^

During the infancy phase, there is a decrease in pulmonary pressures and decline in oxygen levels leading to decreased coronary perfusion and ischemia, especially during feeding or crying when myocardial oxygen demand is increased. Chronic mycardial ischemia leads to impaired function not only of the myocardium, but often also of the mitral valve apparatus with cardiac failure and mitral valve regurgitation following. If this phase is tolerated, then compensatory changes occur over time and the myocardium remodels during in children. Due to the development of intercoronary collaterals from the increasingly large RCA providing collateral supply to the LCA leads to a reversal of flow from the anomalous left coronary into the pulmonary artery.^3^ Finally, there are excessive collateral vessels that lead to the shunting of blood from the RCA via collaterals to the LCA and into pulmonary artery during in adults.^3 , 5^ This is seen as an example of a phenomenon of stealing blood.

Echocardiography is the mainstay of non-invasive diagnostic tool during early screening that depicts the abnormal origin of the LCA with an abnormal jet, left ventricular dilatation, dilated RCA, retrograde filling, the mild hypokinesis of the anterior wall, presence of hyperechogenicity of the endocardium and/or papillary muscles, the RCA diameter as a ratio of the aorta ring diameter, etc.^4^

The diagnosis of ALCAPA has traditionally been made by coronary angiography. In recent years, coronary computed tomographic angiography (CCTA) has emerged as the standard of reference for identification and characterization of coronary artery anomalies. CCTA allows a non-invasive accurate diagnosis, depicting the origin and course of the coronary arteries. Additionally, it offers a three-dimensional assessment of the anatomic relations between coronary arteries and adjacent structures,^6^ and it provides sectional views of cardiac structures from various angles. Hence, it could be considered as the imaging modality of choice to noninvasively delineate coronary vessel anatomy. Moreover, it plays an important role in surgical intervention planning, and it may be a valuable postoperative follow-up tool for patients.^7^

The increasing use of cardiac magnetic resonance imaging (MRI) has not only increased the diagnostic yield but also enabled a better assessment of the consequences of myocardial hypoperfusion and associated congenital defects. The presence of left ventricular dilatation, subendocardial scarring, and regional wall motion abnormalities are indicators of chronic ischemia. And the presence of delayed subendocardial enhancement may be seen on cardiac MRI images, which suggests chronic subendocardial ischemia and is considered as a very important sign, particularly in asymptomatic patients.^3^ Surgical correction should be strongly considered if this finding is present. Cardiac MRI has been increasingly utilized in multiple other studies to guide both diagnostic and therapeutic decisions in patients with ALCAPA.

Reimplantation into the aorta is the only true anatomical repair, but the benefits of MVS at the time of ALCAPA operation should be weighed against the effects of prolonged bypass in the setting of an already ischemic left ventricle.^3^ After mitral valvuloplasty/valve replacement and LCA transplantation, they had no symptoms of blood flow steal phenomenon, and myocardial perfusion scintigraphy did not show any ischemic changes. Postoperative echocardiography 7 days after the procedure showed that LCA originates from the aorta with good visualization of the coronary ostia, left atrium and left ventricle became smaller, no MR and ventricular ejection fraction increased to 72%.

Hence, their exercise-induced dyspnea and MR were likely due to coronary artery steal phenomenon from their abnormal origin of a coronary artery. This report highlights the essence of increasing the preoperative diagnosis rate in China remote village. For patients with moderate or significant mitral insufficiency without other apparent causes, with left ventricular dilatation and the possible presence of hyperechogenicity of the endocardium and/or papillary muscles, without good visualization of the coronary ostia (or with suspicion of the anomalous origin or dilation of the coronary artery) were submitted to CTA or cardiac angiotomography.
